# How do psychological characteristics of family members affected by substance use influence quality of life?

**DOI:** 10.1007/s11136-019-02169-x

**Published:** 2019-03-20

**Authors:** John-Kåre Vederhus, Øistein Kristensen, Christine Timko

**Affiliations:** 1 Addiction Unit, Sørlandet Hospital HF, P.B. 416, 4604 Kristiansand, Norway; 2 Department of Veterans Affairs Health Care System, Stanford University Medical Center, Palo Alto, CA USA; 3 Center for Innovation to Implementation, Stanford University Medical Center, Stanford, CA USA

**Keywords:** Substance use disorders, Affected family member, Quality of life, Family functioning, Codependency, Multigroup confirmatory factor analysis, Norway

## Abstract

**Purpose:**

Addiction is a major health stressor for families, representing an under-researched area with important policy implications. The current aim was to validate the Composite Codependency Scale, which captures the psychological characteristics of affected family members, and assess quality of life as mediated by family functioning.

**Methods:**

Close relatives (*n* = 271) of patients in treatment for substance use disorder (SUD) participated in a 4-day psychoeducational program. We also recruited a general population sample (*n* = 393) via an online social media site. Data were analyzed using multigroup confirmatory factor analysis (MGCFA) and a latent regression model. Differences in subscale latent means were applied to ascertain how the scale discriminated the two populations.

**Results:**

MGCFA yielded a shortened, nine-item partial scalar invariant scale (SCCS) that allowed comparison of latent means. The SCCS discriminated between family members and the general population, with family scoring higher on all three scale dimensions. By effect size, family had higher means (mean differences; 95% confidence intervals) for ‘emotional suppression’ (0.48; 0.36–0.59; *p* < 0.001; effect size, 0.92), ‘interpersonal control’ (0.47; 0.36–0.59; *p* < 0.001; effect size, 0.97), and ‘self-sacrifice’ (0.20; 0.10–0.29; *p* < 0.001; effect size, 0.43). Higher SCCS scores were associated with greater family dysfunction (*β* = 1.00, 95% CI 0.63–1.36; *p* < 0.001) and worse quality of life (*β* = − 0.23, 95% CI − 0.30 to − 0.16; *p* < 0.001), confirming the concurrent validity of the SCCS.

**Conclusion:**

When family members of people with addictions had the psychological characteristics of suppressing their emotions, believing they could fix others’ problems, and neglecting their own for others’ needs, they also had more family dysfunction and poorer quality of life. The SCCS offers a valid instrument for addressing the life situation of affected families. This scale can help clinicians focus on family members within health services, especially within SUD treatment services.

**Electronic supplementary material:**

The online version of this article (10.1007/s11136-019-02169-x) contains supplementary material, which is available to authorized users.

## Background

Addiction in a family member is a major health stressor and seriously affects an estimated 100 million people worldwide [1]. In population studies, family members who experience this stressor have a poorer health status and well-being [2]. They are more likely to be diagnosed with substance use, anxiety, and depression disorders themselves and have higher health care costs than family members of persons with other chronic conditions like diabetes or asthma [3, 4]. This group also typically reports greater family dysfunction (e.g., distrust among family members, lack of support, problematic interactions) than those who do not have addiction in their families [5].

In addition to the direct impact on family health of a substance use or addiction disorder, family members are at risk of developing distinctive psychological characteristics that leave them vulnerable to other health problems. These characteristics include a tendency to self-sacrifice by self-restriction such as suppressing feelings to accommodate the relative’s substance use [1, 6]. Given the large scale of the addiction problem and obvious relevance to public health of these experiences of family members, these underlying characteristics and their association with health and well-being require examination. Such efforts would potentially pave the way for family members to be invited more systematically to participate in treatment of patients with SUDs and to gain access to help for themselves [1].

Trying to care for and change the person with the addiction can come at the expense of the caregiver’s physical, emotional, and spiritual well-being, a phenomenon that often has been characterized as “codependence” [7, 8]. In such situations, the family member or members may engage in self-defeating behaviors that damage self-esteem and quality of life (QoL) [9]. We have observed that family members can make sense of their experiences in light of this concept of codependence. In addition, patients who are more motivated for treatment may also be more invested in repairing relationships after discharge. For this reason, helping families understand codependence and its consequences may be helpful for their loved one in SUD treatment [10].

Acknowledging the controversies around the conceptualization of codependence, especially as it relates to victim-blaming, characterizing family members as “enablers,” and emphasizing negative traits [8, 11], we believe that it is important not to “throw the baby out with the bath water.” Some aspects of codependency still offer a useful psychological framework. Based on a thematic analysis of published definitions, Dear et al. clarified the core defining features of codependency: *external focusing* (e.g., focusing one’s attention on the expectations of others), *emotional suppression* (e.g., deliberate suppression or limited conscious awareness of one’s emotions), *interpersonal control* (e.g., enhanced belief in one’s ability to fix other’s people’s problems), and *self-sacrifice* (e.g., neglecting one’s own needs and focus on the needs of others) [6].

Starting with an initial pool comprising 28 items representing these four core defining features, Marks et al. developed a Composite Codependency Scale (CCS) [12]. Their exploratory factor analysis retained 19 items and three sub-factors (the external focusing factor was absorbed by the interpersonal control factor) and treated ‘Codependency’ as the overarching, common factor. As hypothesized, they found that affected family members had significantly higher CCS total scores than a general population sample and that the CCS explained a substantial amount of variance in a number of variables conceptually related to it (e.g., self-esteem, stress, family dysfunction). For future work, these authors recommended a more rigorous test of factorial validity and inclusion of a larger sample of family members because their study involved only 49 members of Codependents Anonymous in Australia.

In Norway, a recent health policy document underscored the importance of focusing on family members within mental health and SUD treatment services [13], yet contained virtually no concrete measures for reaching these goals. Furthermore, being affected by substance use in the family is no longer a possible referral category in the new referral requirement description for interdisciplinary specialized SUD treatment services [14]. Thus, family members can gain access to treatment services primarily if their loved one with the SUD is in treatment and the family is viewed as a possible resource in that treatment. Any advice and guidance on how to best take care of their own health in a stressful life situation then comes as a “side effect” of the patient’s SUD treatment. However, if family members become ill because of this situation, they should be referred to the treatment level and type they need, whether symptoms are somatic or psychiatric or they develop a substance use problem themselves.

In the present study, we used a codependence framework to examine the psychological profile of these family members. Our aim was to validate the CCS and examine its factor structure with a confirmatory factor analysis (CFA). We hypothesized that the CCS would positively discriminate between affected family members and the general population and that family members would score higher on the scale. The scale first had to be tested for measurement equivalence across groups, so we focused on the sub-dimensions of the CCS to examine how family members differed from the general population. Second, we examined the convergent and concurrent validity of the CCS total score and the relationship between the CCS and constructs from the literature that were expected to correlate with the scale. Our prediction was that higher CCS scores would be associated with higher family dysfunction and lower QoL [3, 5].

## Methods

### Participants and procedures

The family sample (*n* = 271) was recruited from February 2016 to June 2017 at a Norwegian addiction treatment unit where close relatives of patients in treatment for SUD participated in a 4-day psychoeducational program. Family members were partners (16%), parents (32%), offspring (21%), and siblings (31%) of the patients. A main aim of this family program was to improve their situation by giving them information regarding addiction, teaching coping skills, and sharing experiences in a group-based format. The program was limited to relatives > 16 years of age. The general population sample (*n* = 393) was recruited via a link to an online survey posted on Facebook groups or spread via Facebook contacts of the first author. The general population data collection was carried out in autumn 2016. The survey was described as pertaining to QoL and how families work together, and as providing data from a general population sample to compare to data from a family member sample. The sole requirement was that the respondents should be > 16 years of age.

### Measures

A paper-based survey was used for the family sample and a web-based survey for the general population sample. Data collection included standard sociodemographic information: age, sex, educational level (completed at least secondary school level, high school level, bachelor’s degree, or more than bachelor’s degree), and whether participants were living with a spouse/partner. In addition, we asked whether the participants had grown up in a family where at least one parent had serious problem with drug or alcohol use.

#### Composite Codependency Scale (CCS)

The CCS was translated into Norwegian using standard procedures, which included two forward and two backward translations, pretesting, and consultation with the questionnaire’s developer [12, 15]. The scale consisted of 19 statements representing three core defining dimensions of codependency: *self-sacrifice* (six items, e.g., “I always put the needs of my family before my own needs”); *interpersonal control* (seven items, e.g., “I feel that without my effort and attention, everything would fall apart”); and *emotional suppression* (six items, e.g., “Feelings often build up inside me that I do not express”) [12; Table 1]. Participants indicated the extent to which they agreed with each statement on a scale of 1 (strongly disagree) to 5 (strongly agree). In accordance with the underlying theory and the previous validation study of the CSS, we handled the items as reflective indicators of each sub-factor and treated ‘codependency’ as the overarching (second order) common factor. The 19-item total scale and subscales have shown good internal consistency in previous research, with Cronbach alphas > 0.77 [12].

**Table 1:** Composite Codependency Scale (CCS)

	Items	Sub-factor
C1	Because it is selfish, I cannot put my own needs before the needs of others	SS
C2^b^	I try to control events and people through helplessness, guilt, coercion, threats, advice-giving, manipulation, or domination	IC
C3^b^	It makes me uncomfortable to share my feelings with others	ES
C4^b^	It is my responsibility to devote my energies to helping loved ones solve their problems	SS
C5^b^	What I feel isn’t important as long as those I love are okay	SS
C6^b^	I feel compelled or forced to help people solve their problems (i.e., offering advice)	IC
C7^a,b^	I am very open with others about my feelings, no matter what they are (R)	ES
C8^b^	I keep my feelings to myself and put up a good front	ES
C9	I push painful thoughts and feelings out of my awareness	ES
C10^a,b^	My mood is fairly stable and unaffected by the problems and moods of those close to me (R)	IC
C11^b^	I try to control events and how other people should behave	IC
C12	Feelings often build up inside me that I do not express	ES
C13	I always put the needs of my family before my own needs	SS
C14^b^	No matter what happens, the family always comes first	SS
C15	I become afraid to let other people be who they are and allow events to happen naturally	IC
C16	I often put the needs of others ahead of my own	SS
C17	I feel that without my effort and attention, everything would fall apart	IC
C18	I live too much by other people’s standards	IC
C19	I keep my emotions under tight control	ES

*SS* self-sacrifice subscale, *IC* interpersonal control subscale, *ES* emotional suppression subscale

Marks et al. [12]

Ingress statement: “Please indicate the extent to which you agree with each of the following statements on a scale of 1 (strongly disagree) to 5 (strongly agree).”

^a^Item was reverse-scored

^b^Marked questions were excluded in the multigroup confirmatory factor analysis because of the development of the baseline model, resulting in a shortened version of the scale (SCCS)

#### Family dysfunction

We measured family functioning with the general family functioning subscale (GFFS) from the McMaster Family Assessment Device, previously translated and used in a Norwegian context [16]. Respondents complete 12 items answered on a four-point scale (1 = strongly agree, 4 = strongly disagree). Half of the items are negatively formulated (unhealthy functioning items); these items were reversed and a mean score was computed. A higher score indicated greater family dysfunction. Previous studies have reported that the GFFS is internally consistent, and a validation study yielded a Cronbach’s *α* of 0.86 [17]. That study found a mean score of 1.75 in a general population sample and a score > 2.2 was considered indicative of family dysfunction.

#### QoL

Overall QoL was measured with the QoL-5, a five-item generic (non-disease-specific) scale covering perceived mental and physical health, quality of the relationship with important others (partner and friends), and existential QoL, meaning, relationship to self [18, 19]. Responses are scored on a five-point scale ranging from very poor to very good QoL and then recoded into a decimal scale from 0.1 to 0.9, where 0.9 is the highest/best score and 0.1 the lowest/worst [20]. A mean score of 0.7 is considered normative for a general population sample. A minimal clinically important difference between groups has been defined as ≥ 0.1 (i.e., a moderate difference), and ≥ 0.2 has been considered as a substantial difference [19]. Because the QoL-5 is a generic measure, it uses indicators where patients report their QoL on broad areas of functioning. Although simple inspection cannot clarify whether these indicators are reflective or formative, Fayers et al. argued that it is acceptable to consider such indicators as reflective [21]. In the present analysis, we treated the QoL-5 in accordance with that view. The internal consistency of the scale has been reported as good; a previous study found a Cronbach’s alpha of 0.75 [19].

### Statistical analyses

We wanted to compare latent means between groups that had diverse life experiences, so we checked whether the questionnaire measured the constructs in the same way across groups, i.e., that the underlying constructs were invariant (equivalent) across samples [22]. For this purpose, we performed a multigroup CFA (MGCFA). To assess goodness of fit, we used root mean square error of approximation (RMSEA, cut-off value for a good model fit < 0.06, acceptable fit < 0.08), the comparative fit index (CFI, cut-off value for a good fit was > 0.95 and acceptable fit > 0.90), and the standardized root mean squared residual (SRMR, value for acceptable fit is < 0.08) [23, 24].

Baseline first-order models were established separately per group. As noted, we treated the questions as reflective indicators of their respective construct, indicating that they are exchangeable [25]. Thus, to find the most parsimonious model, we removed problematic items that did not work well in the analysis [e.g., had low factor loadings (< 0.4), cross-loadings to non-intended factors, or high error correlation with other items as indicated by the modification indices (MIs)] [25, 26]. Proceeding to the simultaneous MGCFA, a configural model was established to examine if the scale had strong measurement invariance (scalar equivalence) across groups, which is ideal for comparing means [27]. Scalar equivalence implies that the scales have not only the same factor structure (i.e., configural equivalence) and equal factor loadings (i.e., metric equivalence) across groups but also invariant intercepts, i.e., equivalent origin on the scales in the different groups [28]. Cross-group equality constraints on measurement parameters were implemented, and more restricted models were compared with less restricted models (nested models) [29]. Chi-square difference tests between the nested models were applied in which the difference in Chi-square value (Δ*χ*^2^) relative to the change in degrees of freedom (∆*df*) was evaluated, as were changes in CFI (∆CFI) [29]. A non-significant Δ*χ*^2^ value or a ∆CFI ≤ − 0.01 indicated that constraining the parameters did not significantly worsen the fit of the model, and the null hypothesis of measurement invariance could be retained [30].

If the model proved to be not scalar invariant, intercepts of the non-invariant variables would be freed to assume partial scalar equivalence, which is still considered sufficient for comparing latent means if at least two items are metrically invariant per factor [27]. We examined differences in latent means with the ‘general population’ as the reference group. Effect sizes are not directly computed in the program we used (Mplus), so to examine the magnitude of differences in latent means, we calculated an effect size *d* for these differences, following the procedure proposed by Hancock [31]. Common standards for small, medium, and large standardized effects are 0.2, 0.5, and 0.8, respectively [32]. Internal consistency of the scale was reported with the composite reliability (CR) value [33], and a CR value of > 0.70 is considered acceptable.

Convergent validity of the CCS was examined with zero-order correlation analyses, reported with Pearson’s *r*. Finally, we examined the influence of the CCS on family function and QoL in a simultaneous latent regression model. In addition to evaluating the direct effect of the CCS, this procedure allows for the examination of its indirect effect on QoL via the family functioning factor (with the ‘Model Indirect’ syntax). Results are presented as unstandardized beta coefficients (*β*) with 95% confidence intervals (CIs). The *R*-square (*R*^2^) value was used to assess the percentage of the response variable variation explained by the model. Analyses were undertaken with the software program Mplus, version 7.3, and we used the maximum likelihood estimation with robust standard errors [29]. Significance was set at *p* < 0.05.

## Results

**Fig. 1:**
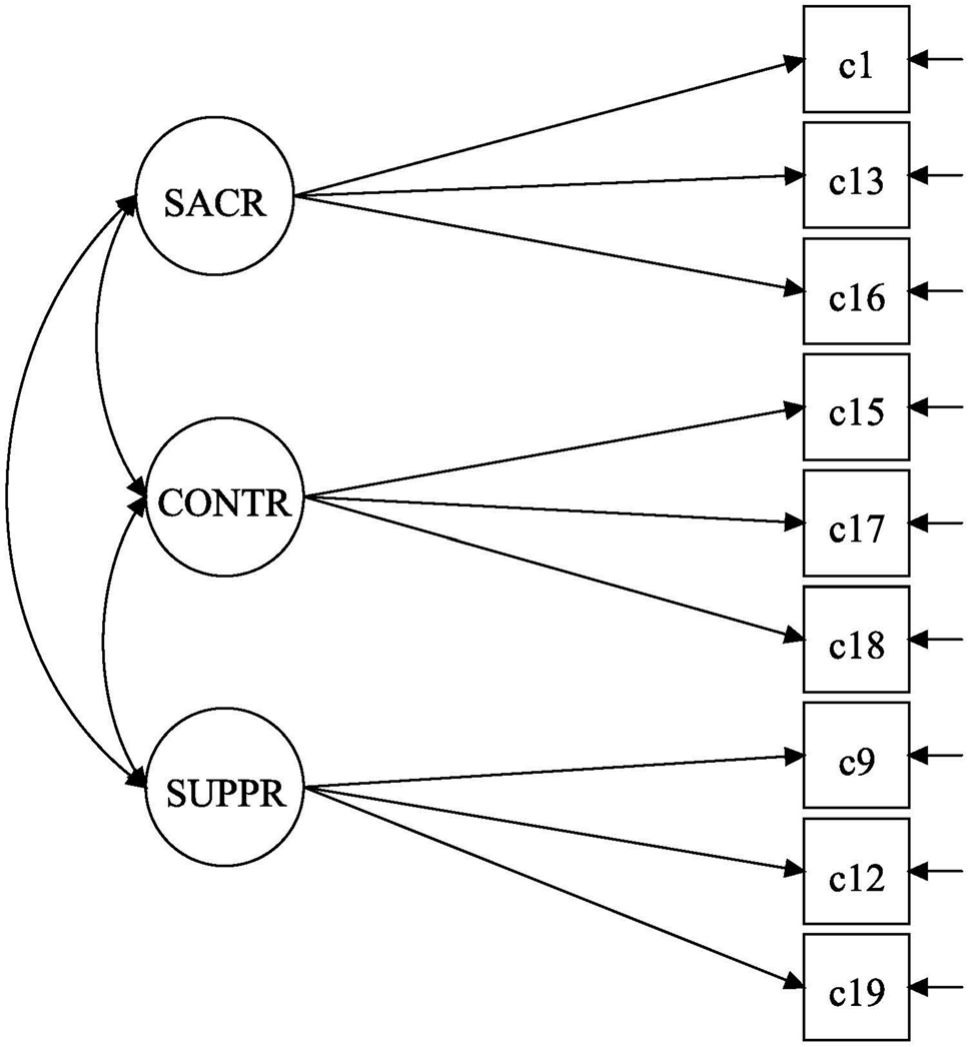
Baseline model for the multigroup confirmatory factor analysis of the Shortened Composite Codependency Scale (SCCS) in the two studied groups: general population sample and affected family members (AFMs). Fit statistics for the baseline model: AFMs—root mean square error of approximation (RMSEA) = 0.04 and comparative fit index (CFI) = 0.97; and general population sample—RMSEA = 0.04 and CFI = 0.98. SACR = self-sacrifice, items C1, C13, C16 in the CCS; CONTR = interpersonal control, items C15, C17, C18 in the CCS; and SUPPR = emotional suppression, items C9, C12, C19 in the CCS

Demographics for the two samples are shown in Table 2. The covariance coverage of the CCS was good, above 98.7% on all items. We have no reason to assume that there was a systematic explanation for the few missing values, and we therefore used the full information maximum likelihood to handle them. Below, we refer to question numbers of the CCS using C and the number (Table 1). The initial model, as described by Marks et al. [12], did not fit the data well; the baseline models had fit indices > 0.08 for RMSEA and < 0.82 for CFI. The MIs indicated several strains in the model with loads of error correlations on items within a factor and across factors. The MI indicates the decrease in model *χ*^2^ statistics if a particular parameter is freed from a constraint; thus, the corresponding fixed parameter should be freed (or item removed) to improve model fit [34]. MIs also indicated several cross-loadings, e.g., that C19, an emotional suppression item, was suggested to load on interpersonal control, and C6, an interpersonal control item, was suggested to load on self-sacrifice. Although it could be argued that some of these adjustments would be reasonable (for example, because of similar item wordings across factors), the amount of adjustment needed discouraged us from this choice. Thus, to find a parsimonious and well-fitting model, we removed problematic items one by one, guided by substantive evaluation of the items and MIs. After these adjustments of the basic model, the baseline models for the different groups had acceptable goodness-of-fit measures, and the same baseline model (a shortened CCS, designated as SCCS) could be used for both groups (Fig. 1). Substantively, we considered that the remaining indicators represented the constructs well.

**Table 2:** Demographics and mean scoring on the original Composite Codependency Scale (CCS), *N* = 664

	General public (*n* = 393)	Affected family members (*n* = 271)
Age (years)	*46* (*12*)	*43* (*16*)
Sex (female %)	301 (77)	178 (66)
Educational level (*n* = 659)		
Higher education (at least bachelor’s degree)	292 (75%)	80 (30%)
Not living with a spouse/partner	66 (17%)	51 (19%)
Adult child of a person with substance misuse	68 (17%)	96 (36%)
Codependency factors		
Self-sacrifice^a^	*3.20* (*0.64*)	*3.44* (*0.64*)
Interpersonal control^a^	*2.61* (*0.77*)	*3.07* (*0.77*)
Emotional suppression^a^	*2.40* (*0.62*)	*2.75* (*0.60*)

Italic variables are shown as means (standard deviation) or n (%)

^a^Mean score on the original version of the scale (see Table 1)

The simultaneous MGCFA indicated that the model was metric equivalent; the corrected *χ*^2^ difference test between the configural and metric model was non-significant (∆*χ*^2^/∆*df* = 6/6, P = 0.37), and the ∆CFI was < − 0.01 (Table 3). However, the model was not scalar equivalent because the corrected difference test between the metric and scalar models was significant (∆*χ*^2^/∆*df* = 16/6, *p* = 0.012). The MIs indicated that the intercept of item C18 was non-invariant. When this intercept was freed (i.e., a partial scalar model), the ∆*χ*^2^/∆*df* difference between the partial and metric models indicated an equal fit with a corrected ∆*χ*^2^ = 5 and ∆*df* = 5, *p* = 0.44. The model had good fit statistics (RMSEA = 0.04, CFI = 0.98, SRMR = 0.05; Table 3), and latent means could be compared [22].

**Table 3:** Multigroup confirmatory factor analysis results of the measurement invariance tests across the two groups (general population sample and affected family members, *N* = 664)

	*χ*^2^	*df*	RMSEA	CFI	SRMR
Configural model	77	48	0.04	0.98	0.04
Metric model	83	54	0.04	0.98	0.05
Scalar model	99	60	0.04	0.97	0.05
Partial scalar model	88	59	0.04	0.97	0.05

*RMSEA* root mean square error of approximation, *CFI* comparative fit index, *SRMR* standardized root mean squared residual

The differences between groups in the latent means for the three constructs of the SCCS are shown in Table 4. The general population sample served as the reference group. All constructs were significantly different. The largest difference was found in ‘interpersonal control’ (mean for family members was 0.47 higher; *p* < 0.001) and ‘emotional suppression’ (mean 0.48 higher for family members; *p* < 0.001). The effect sizes for the differences were 0.43, 0.97, and 0.92 for ‘self-sacrifice,’ ‘interpersonal control,’ and ‘emotional suppression,’ respectively. Thus, the effect size for the difference in ‘self-sacrifice’ was modest, but for the difference in ‘interpersonal control’ and ‘emotional suppression,’ it was large.

**Table 4:** Differences in latent means between the two groups (general population sample and affected family members) on the Shortened Composite Codependency Scale (SCCS), *N* = 664

	General population sample^a^	Affected family members	95% CI	*p* Value
Self-sacrifice	0.00	0.20 (0.05)	0.10–0.29	< 0.001
Interpersonal control	0.00	0.47 (0.06)	0.36–0.59	< 0.001
Emotional suppression	0.00	0.48 (0.06)	0.36–0.59	< 0.001

Latent means was obtained from a multigroup confirmatory factor analysis with cross-groups partial scalar invariance. The brackets show standard errors

^a^Reference group

**Fig. 2:**
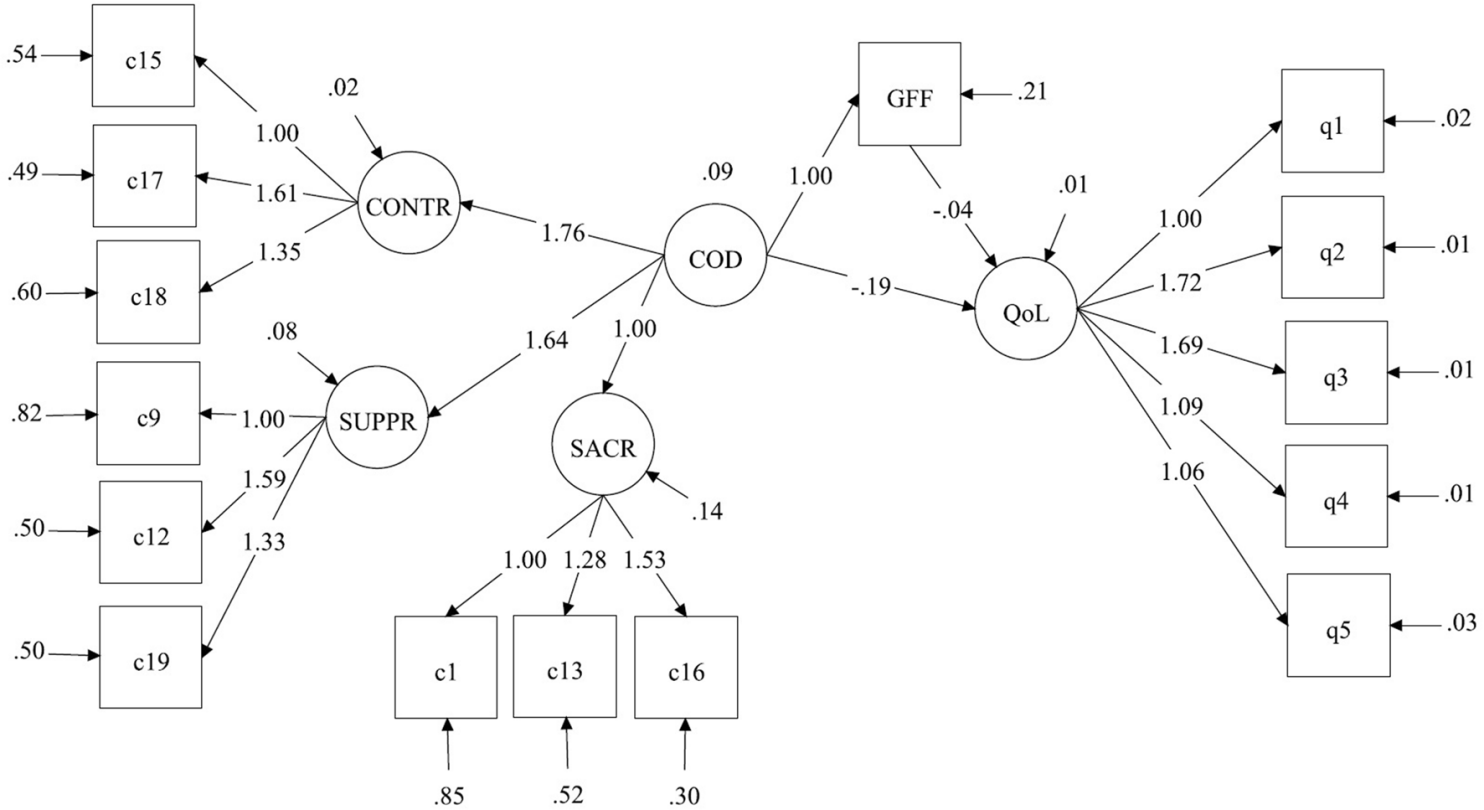
Latent regression analysis to examine the influence of codependence (COD)^a^ on general family functioning (GFF)^b^ and quality of life (QoL)^c^. Parameter estimates are unstandardized. SACR = self-sacrifice, items C1, C13, C16 in the *Composite Codependency Scale* (CCS); CONTR = interpersonal control, items C15, C17, C18 in the CCS; and SUPPR = emotional suppression, items C9, C12, C19 in the CCS. Notes ^a^Measured with the *Shortened Composite Codependency Scale* (*SCCS*). ^b^Mean score of the general family functioning scale (GFFS) of the McMaster Family Assessment Device. ^c^Measured with the QoL-5 scale, items q1–q5

In readying for the latent regression analysis, we discovered that the 12-item GFFS had unacceptable fit statistics when used as a latent construct. The aim of the present study did not involve validating the GFFS, so we chose a conservative approach and used the mean score in the analysis. The scale reliability was excellent (CR = 0.90). Descriptively, the family members reported greater familial dysfunction than the general population, with a score of 2.21 versus 1.90 (a 0.31 higher mean score; 95% CI 0.23–0.39; *p* < 0.001) on the GFFS. In a preliminary step to examine convergent validity, the SCCS was correlated with the GFFS in the expected direction (*r* = 0.49, *p* < 0.001). The regression model showed that a higher SCCS total score was associated with greater family dysfunction (*β* = 1.00, 95% CI 0.63–1.36; *p* < 0.001), so that a one-point higher score on the SCCS scale predicted a one-point higher score on the GFFS and explained 29% of the variance (Fig. 2).

The QoL-5 scale had high construct reliability (CR = 0.84). The SCCS was correlated with the QoL-5 in the expected direction (*r* = − 0.54, *p* < 0.001), which provided support for the scale’s convergent validity. Although the family members had significantly lower QoL compared to the general population sample (0.65 vs. 0.71; mean difference, − 0.06; 95% CI − 0.04 to − 0.08; *p* < 0.001), it was not deemed a clinically important difference at the group level. Nonetheless, in the latent regression analysis, a higher SCCS score had a strong negative influence on QoL. The total effect was *β* = − 0.23 (95% CI − 0.30 to − 0.16; *p* < 0.001), of which β = − 0.19 (95% CI − 0.25 to − 0.13; *p* < 0.001) was a direct and *β* = − 0.04 (95% CI − 0.06 to − 0.02; *p* < 0.001) an indirect effect via the family functioning factor (Fig. 2). In other words, a one-point higher score on the SCCS led to a 0.23-point lower QoL score, a clinically important and substantial influence.

The model explained 51% of the variation in QoL, and the fit of the model was good (RMSEA = 0.05, CFI = 0.95, SRMR = 0.04) (Fig. 2). To control for any potentially confounding effects that sociodemographic covariates may have had (sex, age, education, living arrangement, being an adult child of a parent with substance use problems), these variables were entered into the model (not shown here due to model complexity). When these parameters were added, the fit of the model decreased slightly but was still acceptable (RMSEA = 0.05, CFI = 0.90, SRMR = 0.08). After controlling for these covariates, the effect of SCCS was slightly reduced but still exhibited a substantial influence with *β* = 0.98 (*R*^2^ = 0.29) and *β* = − 0.17 (*R*^2^ = 0.49) on family functioning and QoL, respectively. Concerning the internal consistency of the SCCS in these analyses, the reliability of the latent common factor was high (CR = 0.87), and the reliability for the subscales was acceptable (CR values ranged from 0.68 to 0.73).

## Discussion

Although a considerable amount of adjustment to the CCS was required for this analysis, the present validation of the measure was successful and resulted in a shortened, 9-item version of the scale (SCCS). The family member group scored higher than the general population sample on all measured codependency factors. However, the difference was substantial only in terms of higher ‘emotional suppression’ and ‘interpersonal control,’ based on effect sizes. Higher SCCS total scores had a substantial negative association with QoL, partly because of a direct effect and partly mediated via a negative influence of codependency on family functioning.

The present study builds on a previous exploratory factor analysis of the CCS and contributes further to the literature on how family members are affected by substance use and addiction in their family. By using a latent CFA, as the authors recommended in the original study [12], we applied a more rigorous framework to test whether the scale was in accordance with the underlying theory. The equality of meaning of measured items is usually assumed between groups but not tested. With the MGCFA procedure, we ensured that the factor structure was invariant across groups, justifying latent mean comparisons [35].

The analysis showed that the SCCS discriminated between family members and a general population sample and supported our hypotheses that family members would score higher on the subscales of the SCCS. According to the effect-size evaluation, the difference between groups was modest for self-sacrifice (willingness to sacrifice oneself for loved ones), i.e., significant but not substantial. In contrast, for emotional suppression and interpersonal control (the tendency to be preoccupied with controlling people and situations), differences between family members and the general population sample were substantial. The heightened level of emotional suppression and interpersonal control is likely an underlying reason for the poorer mental health of family members compared with the general population [3].

The SCCS also has concurrent validity. The greater family dysfunction and lower QoL among family members than in the general population group are typical [2, 3, 5]. As hypothesized, higher codependency scores (SCCS total scores) had a negative association with both family functioning and QoL and predicted significant additional unique variance beyond that attributed to sociodemographic variables. The association between SCCS score and these family dysfunction and QoL outcomes also tells us that the SCCS can identify who is more or less psychologically affected by their difficult life situation.

Future research should examine the temporal stability of the SCCS, such as whether its factors are stable constructs or can be influenced by interventions designed to address them. It would also be interesting to use the SCCS in studies examining whether the characteristics it measures are specific to families with a member who has SUD or whether these factors also are elevated when the family stressor is, for example, a serious mental health disorder [36]. A possible hypothesis is that the psychological characteristics that the SCCS measures are somewhat generic and can be viewed as psychological consequences of living with a stigmatized problem within a family. Previous studies have found that family stigma related to drug dependence is worse than for most other health conditions, with family members being blamed for the relative’s disorder [37]. Although families also receive blame to a lesser extent for mental disorders (e.g., schizophrenia), we believe that the SCCS could potentially be relevant for other within-family problems that tend to isolate family members and deprive them of needed social support [37].

### Methodological considerations

The general population sample was a convenience sample, and some differences from the adult Norwegian population as a whole were evident. For example, 75% of the general sample had at least a bachelor’s degree compared to only 33% in the adult Norwegian population [38]. One could argue that this difference between groups limits the generalizability of the comparison. However, although educational level may influence attitudes and opinions, it is less likely to substantially influence psychological characteristics arising from familial stressors. Such stressors exist in every strata of the population. In addition, our regression model controlled for sociodemographic covariates, including education, which did not alter the main finding concerning the association of the SCCS with the dependent factor (QoL).

The general population sample also included a proportion (17%) who were adult children of a parent with substance use problems. No data exist on how many Norwegian adults have personal experience with problematic substance use within their families while growing up, but estimates are that about 10% of people < 16 years old are in a household where at least one parent uses alcohol harmfully [2, 39]. The fact that the present study likely includes a sample with these personal experiences strengthens the generalizability of the findings. The proportion of adults who had personal experience with problematic substance use within their family was higher in the family member sample (36%), which is consistent with previous research and does not invalidate the present comparison [40]. The family sample consisted of family members attending a psychoeducational program, so the results need replication among family members who are not receiving this kind of service.

Although some investigators have hypothesized that social desirability may enhance measured well-being, studies show that any such associations are modest [41]. Nevertheless, we cannot rule out that social desirability influenced participant responses. Specifically regarding the emotional suppression subscale, higher scores could represent greater self-awareness rather than emotional suppression per se; however, the items on the subscale are similar to those on commonly used and well-validated measures of cognitive avoidance coping [42]. Finally, the invariance testing of the CCS accounted for the functioning of the scale across groups and across different data-collection methods [43].

## Conclusions

Overall, our findings indicate that the shortened SCCS is a promising measure in this area of research on family members of people with addiction or other harmful substance use. We found that when family members have a higher codependency score, they also have more family dysfunction and poorer quality of life. The SCCS distinguishes among those more or less affected psychologically by their difficult life situation. Thus, it could be a valuable tool in clinical work and in research focused on affected family members within health care services. The SCCS can potentially also be used within the broader mental health area of research and not limited only to SUD treatment services.

## Electronic supplementary material

Below is the link to the electronic supplementary material.

Supplementary material 1 (CSV 100 KB)

Supplementary material 2 (INP 755 Bytes)

Supplementary material 3 (INP 791 Bytes)

## Data Availability

The dataset and Mplus input files have been uploaded as electronic supplementary material.

## References

[1] Orford J, Velleman R, Natera G, Templeton L, Copello A (2013). Addiction in the family is a major but neglected contributor to the global burden of adult ill-health. Social Science & Medicine.

[2] Casswell S, You RQ, Huckle T (2011). Alcohol’s harm to others: Reduced wellbeing and health status for those with heavy drinkers in their lives. Addiction.

[3] Dawson DA, Grant BF, Chou SP, Stinson FS (2007). The impact of partner alcohol problems on women’s physical and mental health. Journal of Studies on Alcohol and Drugs.

[4] Ray GT, Mertens JR, Weisner C (2009). Family members of people with alcohol or drug dependence: Health problems and medical cost compared to family members of people with diabetes and asthma. Addiction.

[5] Cullen J, Carr A, Id (1999). Carr AOhoo: Codependency: An empirical study from a systemic perspective. Contemporary Family Therapy: An International Journal.

[6] Dear EG, Roberts CM, Lange L, Shohov SP (2005). Defining codependency: A thematic analysis of published definitions. Advances in Psychology Research.

[7] Beattie M (1992). Codependent no more: How to stop controlling others and start caring for yourself.

[8] Granello DH, Beamish PM (1998). Reconceptualizing codependency in women: A sense of connectedness, not pathology. Journal of Mental Health Counseling.

[9] Timko C, Young LB, Moos RH (2012). Al-anon family groups: Origins, conceptual basis, outcomes, and research opportunities. Journal of Groups in Addiction & Recovery.

[10] Caputo A (2018). The experience of therapeutic community: Emotional and motivational dynamics of people with drug addiction following rehabilitation. International Journal of Mental Health and Addiction.

[11] Springer CA, Britt TW, Schlenker BR (1998). Codependency: Clarifying the construct. Journal of Mental Health Counseling.

[12] Marks ADG, Blore RL, Hine DW, Dear GE (2012). Development and validation of a revised measure of codependency. Australian Journal of Psychology.

[13] Ministry of Health and Care Services: Public Helth Report (2015). Coping and possibilities [Folkehelsemeldingen: Mestring og muligheter]. MeldSt 19(2014–2015).

[14] Helsedirektoratet (2015). Priority guidelines for substance use disorder treatment [Prioriteringsveileder—Tverrfaglig spesialisert rusbehandling].

[15] Beaton DE, Bombardier C, Guillemin F, Ferraz MB (2000). Guidelines for the process of cross-cultural adaptation of self-report measures. Spine.

[16] Reigstad B, Jorgensen K, Sund AM, Wichstrom L (2010). Prevalences and correlates of sleep problems among adolescents in specialty mental health services and in the community: What differs?. Nordic Journal of Psychiatry.

[17] Byles J, Byrne C, Boyle MH, Offord DR (1988). Ontario Child Health Study: Reliability and validity of the general functioning subscale of the mcmaster family assessment device. Family Process.

[18] Lindholt JS, Ventegodt S, Henneberg EW (2002). Development and validation of QoL5 for clinical databases. A short, global and generic questionnaire based on an integrated theory of the quality of life. European Journal of Surgery.

[19] Vederhus JK, Birkeland B, Clausen T (2016). Perceived quality of life, 6 months after detoxification: Is abstinence a modifying factor?. Quality of Life Research.

[20] Ventegodt S, Merrick J, Andersen NJ (2003). Measurement of quality of life II. From the philosophy of life to science. ScientificWorld Journal.

[21] Fayers PM, Hand DJ (2002). Causal variables, indicator variables and measurement scales: An example from quality of life. Journal of the Royal Statistical Society.

[22] Byrne BM, Shavelson RJ, Muthen B (1989). Testing for the equivalence of factor covariance and mean structures: The issue of partial measurement invariance. Psychological Bulletin.

[23] Hu L, Bentler PM (1999). Cutoff criteria for fit indexes in covariance structure analysis: Conventional criteria versus new alternatives. Structural Equation Modeling.

[24] Little TD (2013). Longitudinal structural equation modeling.

[25] Brown TA (2006). Confirmatory factor analysis for applied research.

[26] Stevens JP (2009). Applied multivariate statistics for the social sciences.

[27] Steenkamp JEM, Baumgartner H (1998). Assessing measurement invariance in cross-national consumer research. Journal of Consumer Research.

[28] Steinmetz H, Schmidt P, Tina-Booh A, Wieczorek S, Schwartz S (2009). Testing measurement invariance using multigroup CFA: Differences between educational groups in human values measurement. Quality & Quantity.

[29] Muthén LK, Muthén BO (2017). Mplus. statistical analysis with latent variables—user’s guide.

[30] Cheung GW, Rensvold RB (2002). Evaluating goodness-of-fit indexes for testing measurement invariance. Structural Equation Modeling.

[31] Hancock GR (2001). Effect size, power, and sample size determination for structured means modeling and MIMIC approaches to between-groups hypothesis testing of means on a single latent construct. Psychometrika.

[32] Cohen J (1988). Statistical power analysis for the behavioral sciences.

[33] Peterson RA, Kim Y (2013). On the relationship between coefficient alpha and composite reliability. Journal of Applied Psychology.

[34] Brown TA (2015). Confirmatory factor analysis for applied research.

[35] Davidov E, Meuleman B, Cieciuch J, Schmidt P, Billiet J (2014). Measurement equivalence in cross-national research. Annual Review of Sociology.

[36] Birkeland B, Weimand BM, Ruud T, Hoie MM, Vederhus JK (2017). Perceived quality of life in partners of patients undergoing treatment in somatic health, mental health, or substance use disorder units: A cross-sectional study. Health and Quality of Life Outcomes.

[37] Corrigan PW, Watson AC, Miller FE (2006). Blame, shame, and contamination: The impact of mental illness and drug dependence stigma on family members. J Fam Psychol.

[38] Educational level in the Norwegian population. Retrieved February 1 2019 from https://www.ssb.no/utdanning/statistikker/utniv.

[39] Rossow I, Natvig H, Moan IS (2009). Close relatives of persons with harmful use of alcohol—how many are they and how are they affected? [Nære pårørende av alkoholmisbrukere: hvor mange er de og hvordan berøres de?].

[40] Salvatore JE, Larsson Lonn S, Long EC, Sundquist J, Kendler KS, Sundquist K, Edwards AC (2019). Parental alcohol use disorder and offspring marital outcomes. Addiction.

[41] Caputo A (2017). Social desirability bias in self-reported well-being measures: Evidence from an online survey. Universitas Psychologica.

[42] Holahan CJ, Moos RH, Holahan CK, Brennan PL, Schutte KK (2005). Stress generation, avoidance coping, and depressive symptoms: A 10-year model. Journal of Consulting and Clinical Psychology.

[43] Gordoni G, Schmidt P, Gordoni Y (2011). Measurement invariance across face-to-face and telephone modes: The case of minority-status collectivistic-oriented groups. International Journal of Public Opinion Research.

